# Draft Genome Sequence of *Bacillus velezensis* GF610, a Producer of Potent Anti-*Listeria* Agents

**DOI:** 10.1128/genomeA.01046-17

**Published:** 2017-10-12

**Authors:** Michelle M. Gerst, Edward G. Dudley, Lingzi Xiaoli, Ahmed E. Yousef

**Affiliations:** a Department of Microbiology, The Ohio State University, Columbus, Ohio, USA; b Department of Food Science and Technology, The Ohio State University, Columbus, Ohio, USA; c Department of Food Science, Pennsylvania State University, University Park, Pennsylvania, USA

## Abstract

*Bacillus velezensis* GF610 was isolated from soil in Illinois, USA, and found to produce amyloliquecidin GF610, a potent two-component antimicrobial peptide. We report here the GF610 strain draft genome sequence, which contains 4.29 Mb and an overall GC content of 45.91%.

## GENOME ANNOUNCEMENT

*Bacillus* species are known to produce potent antimicrobial compounds (1). *Bacillus velezensis* GF610 was isolated from garden soil originating in Dunlap, IL, USA. A bacterial cell extract of *B. velezensis* was effective against *Listeria innocua* and 11 strains of *Listeria monocytogenes* (M. Gerst and A. Yousef, unpublished data). A lantibiotic, amyloliquecidin GF610, was identified in the crude extract using matrix-assisted laser desorption ionization-time of flight mass spectrometry and Fourier transform ion cyclotron resonance mass spectrometry.

The draft genome sequence of *B. velezensis* GF610 was determined using Illumina MiSeq. A total of 918,314 reads were generated and assembled using the SPAdes genome assembler (version 3.10.1). The assembly was reordered using Mauve against the *Bacillus* sp. strain 275 genome from the NCBI database (2). The final assembly consists of 87 contigs, with a maximum size of 332,580 bp and minimum size of 240 bp. The genome was annotated using the NCBI Prokaryotic Genome Annotation Pipeline.

Secondary metabolite gene clusters were analyzed using antiSMASH 4.0.0 (3) and BAGEL3 (4). Both programs identified the amyloliquecidin lantibiotic gene cluster in contig 19. Additionally, antiSMASH identified 16 other secondary-metabolite gene clusters, including 5 nonribosomal peptide synthetases (NRPS), genes associated with terpenes, and one type III polyketide synthetase gene cluster. The secondary metabolite gene clusters were associated with biosynthesis of difficidin (93% similarity), macrolactin (100% similarity), fengycin (80% similarity), bacillaene (92% similarity), locillomycin (35% similarity), surfactin (52% similarity in contig 4, 47% similarity in contig 10), bacillibactin (84% similarity), and bacilysin (85% similarity). Contigs 13 and 16 contain genes coding for terpene biosynthesis. In addition to the lantibiotic gene cluster for amyloliquecidin GF610, BAGEL3 identified two gene clusters for unmodified bacteriocins, including the gene cluster for the LCI antimicrobial protein. *Bacillus* spp. are known to produce these compounds. For example, *B. velezensis* LM2303 produces surfactin, iturin, fengycin, bacillaene, and macrolactin (5). Strains of *Bacillus amyloliquefaciens* were found to produce difficidin, macrolactin, fengycin, bacillaene, surfactin, bacillibactin, and bacilysin (6–10). Locillomycin is produced by *Bacillus subtilis* (11).

The lantibiotic gene cluster in contig 19 was analyzed via the National Center for Biotechnology Open Reading Frame Finder (12). The open reading frames necessary for the production of a two-component lantibiotic were identified. The gene cluster included those for amyloliquecidin GF610 alpha and beta structural gene peptides (*lanA1* and *lanA2*), two *lanM*-type modification enzymes, an ABC transporter gene (*lanT*), six additional ABC transporter genes (*lanFEG1* and *lanFEG2*), a protease (*lanP*), and a response regulator. This gene cluster is similar to that in *B. amyloliquefaciens* AD 2, which was isolated in South Africa and produced amyloliquecidin AD 2 (13). The lantibiotic gene cluster identified in the draft genome of *B. velezensis* GF610 helped elucidate the structure and identity of a potent two-component anti-*Listeria* metabolite. Additionally, this draft genome is the first description of the amyloliquecidin gene cluster in *B. velezensis* and on the American continents.

### Accession number(s).

The *Bacillus velezensis* GF610 whole-genome sequence has been deposited in NCBI/GenBank database and assigned the accession no. NQXV00000000. The version described in this paper is the first version, NQXV01000000.
